# The Heterogeneity of Early Parkinson’s Disease: A Cluster Analysis on Newly Diagnosed Untreated Patients

**DOI:** 10.1371/journal.pone.0070244

**Published:** 2013-08-01

**Authors:** Roberto Erro, Carmine Vitale, Marianna Amboni, Marina Picillo, Marcello Moccia, Katia Longo, Gabriella Santangelo, Anna De Rosa, Roberto Allocca, Flavio Giordano, Giuseppe Orefice, Giuseppe De Michele, Lucio Santoro, Maria Teresa Pellecchia, Paolo Barone

**Affiliations:** 1 Sobell Department of Motor Neuroscience and Movement Disorders, University College London (UCL), London, United Kingdom; 2 IDC Hermitage - Capodimonte, Naples, Italy; 3 University Parthenope, Naples, Italy; 4 University Federico II, Department of Neurological Science, Naples, Italy; 5 Neuropsychology Laboratory, Department of Psychology, Second University of Naples, Caserta, Italy; 6 University of Salerno, Center for Neurodegenerative Diseases - CEMAND, Salerno, Italy; University of Chicago, United States of America

## Abstract

**Background:**

The variability in the clinical phenotype of Parkinson’s disease seems to suggest the existence of several subtypes of the disease. To test this hypothesis we performed a cluster analysis using data assessing both motor and non-motor symptoms in a large cohort of newly diagnosed untreated PD patients.

**Methods:**

We collected data on demographic, motor, and the whole complex of non-motor symptoms from 100 consecutive newly diagnosed untreated outpatients. Statistical cluster analysis allowed the identification of different subgroups, which have been subsequently explored.

**Results:**

The data driven approach identified four distinct groups of patients, we have labeled: 1) Benign Pure Motor; 2) Benign mixed Motor-Non-Motor; 3) Non-Motor Dominant; and 4) Motor Dominant.

**Conclusion:**

Our results confirmed the existence of different subgroups of early PD patients. Cluster analysis revealed the presence of distinct subtypes of patients profiled according to the relevance of both motor and non-motor symptoms. Identification of such subtypes may have important implications for generating pathogenetic hypotheses and therapeutic strategies.

## Introduction

Parkinson’s disease (PD) clearly manifests a heterogeneous clinical syndrome and this variability in the clinical phenotype seems to suggest the existence of several subtypes of the disease [1]. Assuming that homogeneous groups of patients are more likely to share pathological and genetic features, recognition of different subgroups of patients may be relevant for research on underlying pathophysiology, with crucial consequences for our understanding of disease progression, prognosis and treatment strategies.

Subtypes of PD have previously been profiled mainly according to the relevance of such demographic and clinical features as age at disease onset and motor phenotype [2]–[5]. Recently, two independent groups reviewed the results of the cluster analyses performed on PD patients, showing that the cluster profiles “old age-at-onset with rapid disease progression” and “young age-at-onset with slow disease progression” emerged from the majority of studies [6], [7]. Two of the examined studies further identified the “tremor-dominant” and the “bradykinesia/rigidity and PIGD dominant” subgroups [8], [9], while other profiles were less consistently revealed.

Presence of different subgroups of PD patients has been less investigated from a non-motor viewpoint [1], [6]–[8]. Cognitive dysfunctions, particularly deficits in tasks such as set-shifting, sequencing, and planning (executive functions), have been found to be associated with some motor features including bradykinesia, axial involvement and gait disturbances [10]–[15]. Depression has been consistently reported as one the most frequent psychiatric features in PD and it has been supposed to represent a distinct subtype of disease [16]. Apart from cognitive and psychiatric disturbances, there are only few observations suggesting that non motor symptoms (NMS) may group with either demographic or clinical features in PD [17]–[20].

Moreover, previous research included patients treated with dopaminergic therapy. Dopaminergic therapy has been reported to affect the NMS, including cognition and mood [21]–[23] and this might be a potential confounding factor.

To test the existence of subgroups that may be profiled according to the presence of NMS, we performed a cluster analysis using both motor and non-motor data of a large cohort of newly diagnosed untreated PD patients.

## Patients and Methods

### Ethics Statement

The study was approved by the ethics committee of the University Federico II of Naples, and all patients provided written informed consent.

### Patients and Clinical Evaluation

All the patients included in this study were prospectively enrolled in an ongoing research project conducted at the Movement disorder center, University Federico II of Naples, Italy, between 2008 and 2010.

Inclusion and exclusion criteria have been extensively described elsewhere [24]–[27]. In brief, inclusion criteria were: 1) the presence of a parkinsonian syndrome according to UK Parkinson’s Disease Society Brain Bank Diagnostic Criteria [28]; 2) disease duration less than 2 years; and 3) no history of present or past therapy with anti-parkinsonian agents. Additional criteria for inclusion were lack of significant cerebral lesions on MRI or severe concomitant disease that might explain the presence of neurological or psychiatric disturbances. None of the patients were treated with anti-cholinergic agents, choline esterase inhibitors, antidepressants, anxiolytic drugs, or other centrally acting substances. Detailed clinical informations were obtained from patient’s history and neurological examination. After 1 and 2 years, all patients underwent a clinical follow-up to confirm the diagnosis of PD according to both positive response to dopaminergic therapy and exclusion of atypical symptoms/signs according to the Queen Square Brain Bank criteria for PD [28]. We excluded from the analyses those patients for which the diagnosis of idiopathic PD was not confirmed during the follow-up.

### Motor Data Collection

The Unified Parkinson’s Disease Rating Scale III (UPDRS-III) was used to evaluate motor disability. Side of motor onset and disease duration (from the motor symptoms appearance to the date of the visit) were recorded. This way of defining disease duration can be prone to recall bias. Nevertheless, good agreement has been achieved when comparing medical records with both family- and subject-history questionnaire to establish the time of onset in PD and all three methods have been regarded valid [29]. In order to highlight the presence of different motor phenotypes, we attempted to compute variables from sub-scores of the UPDRS III, as previously suggested [9], [30].

A “Tremor score” was derived as the mean of the sub-scores of items 20 and 21 (rest and action tremor) of UPDRS III. A categorical variable was also introduced to distinguish tremulous and non-tremulous PD patients. A “Bradykinesia score” was defined as the mean of the sub-scores of items 23 to 26 (finger tap, hand grip, pronosupination, leg tap) of UPDRS III. The mean value of items 27 to 31 provided the “axial score” while the progression rate was calculated as UPDRS III/disease duration. Moreover, motor scores obtained from both 1 and 2 year follow-up evaluations were used to provide a more accurate estimate of the progression rate. All patients were tested in off-state after appropriate pharmacological washout and motor data were collected again. L-Dopa equivalent daily doses (LEDD) were calculated as previously described [31]. Using such follow-up data we performed further post-hoc analyses, as detailed in the Statistical analyses section.

### NMS Data Collection

The Mini-Mental State Examination (MMSE) was used to explore global cognition, while the Frontal Assessment Battery (FAB) to focus on frontal dysfunctions. Scores were age- and education-adjusted, according to Italian normative data [32]. Depression and anxiety were assessed by means of the Hospital Anxiety Depression Scale (HADS), depression-subscale (HADS-D) and anxiety-subscale (HADS-A). All patients completed the Non Motor Symptoms Questionnaire (NMSQuest), a validated and recommended tool for detection of NMS in PD [33]. The NMSQuest consists of 9 NMS domains (NMS-D), each of which includes 2 to 7 specific questions with dichotomous (yes/no) answers for a total of 30 items. Patients (and care-givers) were asked to report specific symptoms and domains as “present/absent” as referred to the month before the visit.

### Statistical Analysis

The cluster analysis was performed using baseline demographical, motor- and non-motor- data. Motor data included the total UPDRS III and the variables computed as described above. Non-motor data included the total number of NMS and NMS-D, each NMS-D as well as the scores obtained from the MMSE, FAB, HADS, HADS-D, and HADS-A. These variables were subjected to a non-hierarchical cluster analysis (k-means method) using the Gower method for mixed data (continuous and categorical data), for 3 to 6 clusters solutions. The Calinski-Harabasz pseudo-F value was used to asses when the clustering optimum solution was attained. Post-hoc comparisons of scores were conducted as appropriate: multivariate analysis of variance complemented by Scheffé post hoc tests was used for continuous variables; the chi-square technique, or Fisher’s Exact when expected values were small, with Yate’s correction where relevant, was used for the comparison of categorical data. Finally, an ordered logistic regression using the stepwise option (which is a forward selection allowing elimination) and including all NMS was carried out to assess variables that were explanatory correlates of clusters. In this model, continuous variables were dichotomized to their medians to help interpretation.

Motor data obtained from the follow-up evaluations have been used for further post-hoc analyses, with the aim to test the reliability of the “progression rate” score used for the clustering. Specifically, total UPDRS-III, tremor-, bradykinesia-, and axial-scores have been compared between groups with the ANOVA test for repeated measures. LEDD and proportion of patients on L-dopa were also compared between clusters, using the t-test and the chi-2 test, respectively.

Statistical analyses were done with the STATA software, version 11.0 (StataCorp LP, USA).

## Results

We enrolled 127 de-novo untreated PD patients. At follow-up, we excluded 27 patients from the analysis: 6 patients due to a diagnosis other than PD (namely, 3 Multiple System Atrophy, 1 Progressive Supranuclear Palsy, 1 Cortico-Basal Syndrome, 1 Lewy-body Dementia); 6 patients were not able to perform the follow-up evaluation (3 could not be reached and 3 withdrew their consent); and for 15 patients, data were incomplete. Thus, 100 PD patients were included in the present analysis. A summary of their characteristics is shown in Table 1.

**Table 1:** Demographic and clinical data in the whole cohort of patients.

**Sex (Male/Female)**	59/41
**Age (years)**	59.7±8.3
**Age at onset (years)**	58.4±8.7
**Disease Duration (months)**	13.4±5.6
**UPDRS-III**	15.3±7.4
**Tremor Score**	1.72±1.58
**Bradikynesia Score**	4.98±3.45
**Axial Score**	3.89±2.19
**Progression rate**	1.41±1.12
**MMSE**	27.07±1.98
**FAB**	13.8±1.88
**HADS**	11.49±5.36
**HADS-D**	5.85±3.01
**HADS-A**	5.64±2.94
**Total NMS per patient**	4.3±2.9
**Total NMS-D per patient**	2.93±1.82

Abb. UPDRS-III: Unified Parkinson’s Disease Rating Scale, motor section; MMSE: Mini Mental State Examination; FAB: Frontal Assessment battery; HADS: Hospital Anxiety Depression Scale; HADS-D: Hospital Anxiety Depression Scale-depression subscale; HADS-A: Hospital Anxiety Depression Scale-anxiety subscale; NMS: non-motor symptoms; NMS-D: non-motor domains.

The clustering optimum was attained for the 4 clusters solution (Calinski-Harabasz pseudo-F = 48.53). Table 2A illustrates the mean values of each baseline continuous variable for the four identified clusters, while results of analyses on baseline categorical data are listed in table 3.

**Table 2:** Group characteristics (continuous variables) at baseline (A) and 2-year follow-up (B).

A)	Group 1- BPM(n = 21)	Group 2- bmM-MN (n = 32)	Group 3- NMD(n = 27)	Group 4- MD(n = 20)	F value
Age (years)	55.4±8.6^a^	60.4±7.8	59.1±7.6	63.7±7.9^b^	3.66
Age at onset (years)	54.2±8.5^a^	59.2±8.0	57.8±7.6	62.3±7.9^b^	3.47
Disease duration (months)	12.9±4.9	13.4±5.6	13.7±4.6	13.1±3.4	0.39
UPDRS III	14.1±4.0^d^	9.3±3.7^c^	17.4±3.7^d^	25.4±6.0^c^	57.36
Tremor score	1.9±.1.5	1.2±1.3^a^	2.0±.1.4	2.5±1.9^e^	3.67
Bradykinesia score	3.8±2.1^c^	2.6±1.3^c^	5.5.±2.3^c^	9.9±.2.6^c^	53.04
Axial score	3.4±2.0^d^	2.7±1.4^c^	4.4±1.8^d^	5.9±2.2^c^	20.91
Progression Rate	1.1±.47^d^	.79±.45^c^	1.5±.68^d^	2.7±.73^c^	18.55
MMSE	27.4±1.2	26.9±1.6	26.9±1.5	26.9±2.3	0.29
FAB	14.6±1.5^a^	14.1±1.8	13.2±1.6	12.9±1.9^b^	11.62
HADS	9.5±3.7^a^	10.1±5.1^a^	11.5±4.2^a^	15.4±6.5^c^	6.73
HADS-D	4.2±2.7^a^	5.4±2.8^a^	6.0±2.5^a^	7.8±2.8^c^	4.26
HADS-A	5.1±2.2	4.7±2.3^a^	5.5±2.8	7.5±3.4^e^	10.21
Total NMS	1.3±1.8^c^	4.1±2.1^f^	5.7±2.5^g^	4.7±2.1^g^	12.44
Total NMS-D	1.1±1.0^c^	2.9±2.0^f^	3.8±1.4^c^	2.8±1.2^f^	12.86
**B)**	**Group 1- BPM** **(n = 21)**	**Group 2- bmM-MN** **(n = 32)**	**Group 3- NMD** **(n = 27)**	**Group 4- MD** **(n = 20)**	**–**
UPDRS III	15.1±3.7^d^	9.5±4.1^c^	17.9±4.3^d^	28.4±5.6^c^	–
Tremor score	2.1±.1.3	1.8±2.1	2.2±.1.4	2.6±1.3	–
Bradykinesia score	3.3±2.5	2.7±1.5	5.1.±3.4	10.3±.2.4^c^	–
Axial score	3.5±1.9	2.9±2.1	4.4±2.3	7.4±2.6^c^	–
LEDD	195.3±100.3	180.6±110.5	240±135.6	350.6±168.4^c^	–

Abb. UPDRS-III: Unified Parkinson’s Disease Rating Scale, motor section; MMSE: Mini Mental State Examination; FAB: Frontal Assessment battery; HADS: Hospital Anxiety Depression Scale; HADS-D: Hospital Anxiety Depression Scale-depression subscale; HADS-A: Hospital Anxiety Depression Scale-anxiety subscale; NMS: non-motor symptoms; NMS-D: non-motor domains; LEDD: Levo-dopa equivalent daily dosage.

a different from group 4 (p<0.01).

b different from group 1 (p<0.01).

c different from all groups (p<0.01).

d different from group 2 and 4 (p<0.01).

e different from group 2 (p<0.01).

f different from group 1 and 3 (p<0.01).

g different from group 1 and 2 (p<0.01).

**Table 3:** Group characteristics (baseline categorical data).

	Group 1- BPM (n = 21)	Group 2- bmM-MN (n = 32)	Group 3- NMD (n = 27)	Group 4- MD (n = 20)	p value	Group differing
Gender, male (%)	12 (57.1)	15 (46.8)	14 (51.8)	9 (45)	0.344	
Onset <55y (%)	13 (61.9)	7 (21.8)	10 (37.1)	2 (10)	0.001	1 and 4 vs all
Tremulous phenotype (%)	19 (90.4)	20 (62.5)	21 (77.8)	15 (75)	0.051	
Positive familial history (%)	7 (33.3)	0 (0)	8 (29.6)	2 (10)	0.001	1 and 3 vs 2 and 4
Right side at onset (%)	9 (42.8)	17 (53.1)	13 (48.1)	8 (40)	0.148	
BIlateral involvement (%)	4 (19.1)	4 (12.5)	2 (7.4)	10 (50)	0.001	4
Digestive Domain (%)	6 (28.5)	18 (56.2)	16 (59.2)	9 (45)	0.163	
Urinary Domain (%)	5 (23.8)	4 (12.5)	12 (44.4)	5 (25)	0.04	3
Memory Domain (%)	0 (0)	20 (62.5)	19 (70.3)	9 (45)	0.001	1
Depression/anxiety Domain (%)	4 (19.1)	23 (71.8)	21 (77.8)	13 (65)	0.001	1
Sleep Domain (%)	3 (14.2)	19 (59.4)	17 (62.9)	10 (50)	0.001	1
Sex Domain (%)	2 (9.5)	10 (31.2)	0 (0)	0 (0)	0.001	2
Miscellany (%)	2 (9.5)	8 (25)	10 (37.1)	6 (30)	0.162	
Cardiovascular Domain (%)	1 (4.7)	7 (21.8)	6 (22.2)	2 (10)	0.272	
Delusion/Hallucinations (%)	0 (0)	0 (0)	0 (0)	0 (0)	NA	

### Group 1: Benign Pure Motor

Twenty-one patients (21%) with a mean age of 55.4±8.6 years constituted the group. This group showed the lowest number of NMS and NMS-D compared to all groups (Scheffé post hoc tests, p<0.01). Age and Age at onset, FAB, HADS and HADS-D scores were significantly lower than group 4 (Scheffé post hoc tests, p<0.01). UPDRS III, Axial score, and Progression rate were lower than group 4, but higher than group 2 (Scheffé post hoc tests, p<0.01), while the Bradykinesia score was found to be higher than group 2, but lower than groups 3 and 4 (Scheffé post hoc tests, p<0.05).

### Group 2: Benign Mixed Motor-Non-Motor

Thirty-two patients (32%) with a mean age of 60.4±7.8 years constituted the group. Compared to all other groups, patients included in this cluster showed the lowest UPDRS III, Bradykinesia score, Axial score (Scheffé post hoc tests, p<0.01), and Progression rate (Scheffé post hoc tests, p<0.05), while more frequently referred sexual disturbances (Fisher’s Exact test, p<0.01). There was a negative association between patients included in this group and a positive familial history (Fisher’s Exact test, p<0.01). Compared to group 4, Tremor score, HADS, HADS-D and HADS-A scores were significantly lower (Scheffé post hoc tests, p<0.01). Total number of NMS and NMD were higher than in group 1 (Scheffé post hoc tests, p<0.01), but lower than group 3 (Scheffé post hoc tests, p<0.01).

### Group 3: Non-Motor Dominant

Twenty-seven patients (27%) with a mean age of 59.1±7.6 constituted the group. Compared to all groups, patients clustered in group 3 reported the highest number of NMS-D (Scheffé post hoc tests, p<0.01), and more frequently referred the Urinary domain as affected (Fisher’s Exact test, p<0.01). Compared to group 1 and 2, patients in this cluster showed more NMS (Scheffé post hoc tests, p<0.01). UPDRS III, Axial score and Progression rate were significantly higher than group 2, but lower than group 4, (Scheffé post hoc tests, p<0.01). Bradykinesia score was higher than groups 1 and 2, but lower than group 4 (Scheffé post hoc tests, p<0.01). HADS and HADS-D scores were lower than group 4 (Scheffé post hoc tests, p<0.01).

### Group 4: Motor Dominant

Twenty patients (20%) with a mean age of 63.7±7.9 years constituted the group. Patients grouped in this cluster showed the highest UPDRS III, Bradykinesia and Axial scores, Progression rate, HADS and HADS-D (Scheffé post hoc tests, p<0.01). Group 4 was associated with bilateral involvement (Fisher’s Exact test, p<0.01). Age and Age at onset were higher than in group 1 (Scheffé post hoc tests, p<0.01). There was a negative association between patients included in this group and a positive familial history (Fisher’s Exact test, p<0.01). Tremor score was found to be higher than group 1 (Scheffé post hoc tests, p<0.01). Number of NMS-D were significantly lower than group 3 (Scheffé post hoc tests, p<0.01), but higher than groups 1 (Scheffé post hoc tests, p<0.01). FAB was significantly lower than group 2 (Scheffé post hoc tests, p<0.01).

Table 4 summarizes the characteristics of the four groups of patients identified through the cluster analysis. Data obtained from the 2-year follow up evaluation have been used mainly to test the a priori hypothesis that the “Motor Dominant” subgroup would have progressed faster in terms of motor scores, requiring higher LEDD. Such data are detailed in table 2B. Briefly, “Motor Dominant” group showed a more severe progression of UPDRS III, bradykinesia, and axial scores (Anova test for repeated measures, p<0.01) and required higher LEDD (t-test, p<0.01) compared to all other clusters. Moreover, an higher proportion of patients belonging to this cluster was on L-Dopa, compared to all other groups (9 out of 20, i.e. 45% vs 5%, 3% and 25%, respectively; chi2-test, p<0.01).

**Table 4:** The characteristics of the four subgroups identified.

Group 1 - BPM (n = 21)	Group 2 - bmM-NM (n = 32)	Group 3 - NMD (n = 27)	Group 4 - MD (n = 20)
54 years at onset	59 years at onset	58 years at onset	62 years at onset
Intermediate UPDRS III score (with mild tremor and bradykinesia scores) Intermediate Progression rate	Low UPDRS III score (with low tremorand bradykinesia scores)Low Progression rate	Intermediate UPDRS III score (with intermediate tremor, bradykinesia and axial scores) Intermediate Progression rate	High UPDRS III score (with high bradykinesia and axial scores) High Progression rate
Absent depression, anxiety and frontal cognitive impairment	Mild depression, anxiety and frontal cognitive impairment	Intermediate depression, anxiety and frontal cognitive impairment	High depression, anxiety and frontal cognitive impairment
Very low NMS score (Memory, Sleepand Psychiatric domains selectivelyspared)	Intermediate NMS score (Sex domain selectively affected)	High NMS score (Urinary domain selectively affected)	Intermediate NMS score

The logistic regression showed that UPDRS III (β coefficient = 0.51; 95% CI:[0.36;0.64]; p<0.001), Sex Domain (β coefficient = -5.16; 95% CI:[−7.49; −2.81]; p<0.001) and Acting out during dreams (β coefficient = 1.04; 95% CI:[0.18;2.13]; p<0.05) classified 50% of patients into the correct cluster (pseudo-R^2^ = 0.499).

## Discussion

To the best of our knowledge, this is the first attempt to explore the heterogeneity of early PD using a data driven approach including both motor features and the whole non-motor complex in a large cohort of newly diagnosed untreated patients. To date only one study has been published using a cluster analysis to describe subgroups in early PD and including untreated patients [34]. However, only 30% of their patients were actually untreated and the whole cohort was hospital based, thus hampering generalization of their findings to the general PD population.

**Figure 1:**
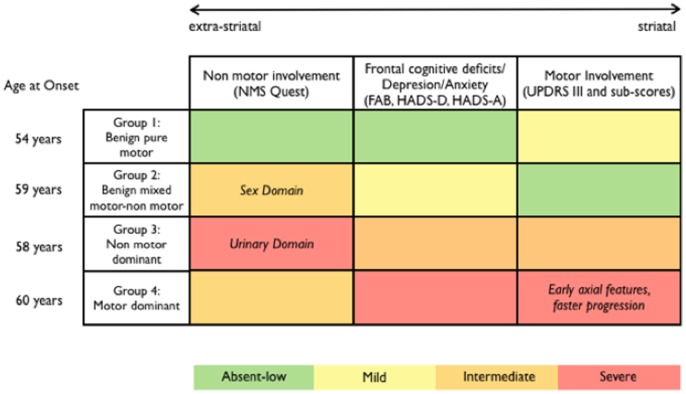
Summary of main features of the clusters according to clinical involvement, severity and age at onset.

Our data driven approach identified four distinct groups of patients, which are delineated by the different involvement of motor and non-motor domains. We have therefore labelled them as Benign Pure Motor (BPM, group 1), Benign Mixed Motor-Non-Motor (bmM-NM, group 2), Non-Motor Dominant (NMD, group 3); and Motor Dominant (MD, group 4). This roughly suggests that there are two benign subtypes (one with prevalent motor impairment and one with prevalent non-motor impairment) and two more severe subtypes of PD (once again, one with prevalent motor impairment and one with prevalent non-motor impairment). Obviously, the term “benign” might be inappropriate for such neurodegenerative diseases as PD. However, it is nowadays clear that PD patients are very heterogenous in terms of progression. For the sake of simplicity, the term “benign” should be intended to recognize those patients with a relative slow short-term progression and possibly longer time span to reach such milestones as motor complications, falls, and dementia [35]. On the other hand, we used the term “dominant” to indicate a prevalent, but not exclusive, feature for the two more severe clusters, which otherwise show an overall higher degree of both motor and non-motor involvement than the two benign groups. Figure 1 highlights differences between clusters. Our results confirmed the existence of two clusters previously described (i.e. “young age-at-onset with mild motor impairment” and “old age-at-onset with rapid disease progression”), but further revealed the presence of two distinct subgroups of patients, which have been profiled according to both presence and relevance of NMS. With this regard, comparisons with previously identified subtypes are not straightforward.

Our results are in agreement with previous studies [1], [6], [8], which consistently identified a subgroup of PD patients with young onset of disease. BPM cluster showed mild motor impairment with predominance of tremor symptoms, slow rate of progression and mild cognitive deficits, as previously reported [1], [6], [9]. Moreover, we also showed that BPM group exhibited the lowest scores of total NMS and NMS-D. This suggests that BPM cluster manifests mild motor features with virtually absent non-motor involvement.

Interestingly, prevalence of patients reporting positive familial history was similar in BPM and NMD clusters, the latter having the second youngest age at onset (57.8±7.6 years) and sharing with the BPM a similar pattern of motor disability. The main difference between these two groups, beyond age at onset, was represented by the NMS, being BPM the group which showed the lowest score of NMS, while NMD group the highest. Indeed, the option to subject NMS to clustering allowed the distinction of these two subgroups which, in previous studies, probably merged together to identify the cluster with younger age at onset, mild motor involvement and rate of progression. Identification of clinical subgroups with almost equivalent motor disability and different non-motor involvement may be crucial, suggesting that independent processes are responsible for motor and non-motor symptoms.

Although BPM group showed a mild motor impairment, we failed to associate it with the best motor performances. Indeed, patients clustering identified a group with 59 years as mean age at onset, showing the lowest motor scores and the slowest rate of disease progression. Two studies have previously found less disability in patients with later symptoms at onset [36], [37], thereby resembling the group we labelled bmM-NM. The latter exhibited an intermediate involvement of the “non-motor system”, placing in between BPM and NMD groups. Interestingly, patients clustered in the bmM-NM complained sexual disturbances more frequently compared to all other groups. Sexual symptoms in PD may be part of autonomic dysfunction in PD and testosterone deficiency has been also implicated [38]. Moreover, sexual disturbances have been reported to increase with motor disability [39], which is not our case. Thus, bmM-NM could represent a less explored group, that may encourage future research, also to test its validity.

The final subgroup identified in our analysis confirmed the existence of a cluster of patients characterized by the highest motor scores and the fastest rate of disease progression [1], [2], [6]–[11]. In line with previous studies, MD cluster showed higher bradykinesia, axial scores, depression, anxiety and frontal impairment than all other groups. It may be argued that higher motor scores in this cluster may be consequential to higher level of depression. However, the findings obtained from the 2-year evaluation suggest that this cluster may have an intrinsic inclination to progress faster, irrespective of mood. Interestingly, there is a body of evidence linking early axial involvement and gait disturbances with frontal cognitive impairment and depression, suggesting a shared underlying mechanism [12], [40]. Moreover, depression [41], [42], anxiety [43] and frontal cognitive impairment [44] have all been supposed to be mediated by disruption of dopaminergic projections to the frontal cortex. Thereby, MD could represent a group with underlying marked dopaminergic degeneration and with relative sparing of extra-dopaminergic systems (intermediate total NMS-D score). With this regard, it should be stressed that the scores measuring total NMS and NMS-D reflect more the involvement of different non-motor domains, rather than an index of their severity. It means that the NMD cluster would have widespread involvement of NMS-D, but milder non-motor severity (at least regarding depression, anxiety and frontal impairment) compared to MD group, possibly suggesting a mild to moderate dopaminergic degeneration (as also confirmed by the intermediate motor scores) and the involvement of extra-dopaminergic systems, which instead would be relatively spared in the MD group. The latter would therefore show an attitude for the involvement of such non-motor features (i.e. frontal-type cognitive deficits and neuropsychiatric issues), which have been consistently linked to the striatal dopaminergic denervation [12], [40]–[44], whereas the NMD cluster would have a widespread involvement of several NMS-D, with possibly further underpinning mechanisms.

One would suspect some NMS-D such as urinary, gastrointestinal and cardiovascular (i.e. all domains which have been to supposed to be part of the autonomic system) to travel together. We failed to identify clear patterns of non-motor grouping in such sense. A limitation which may accounts for this is that the NMSQuest simply detects the involvement of different domains, including such as the gastrointestinal, which may be not specific for PD. Moreover, by considering disaggregated items according to their own relevance (i.e., not the raw number of gastrointestinal symptoms but a measure of the intensity of each one of them), it may be possible to disclose more delineated non-motor grouping. The relative low frequency of some NMS (due to the nature of our cohort of de-novo patients) may have further accounted for such lack of non-motor grouping. Nevertheless, we found clear non-motor differences between groups. For instance, NMD is characterized by urinary issues while MD is characterized by cognitive/neuropsychiatric symptoms, suggesting that these two NMS-D travel separately, in line with other reports [45]. It may further indicate that such two groups (i.e., the “advanced” clusters, which to some extent share a common pattern of motor disability) may be prone to develop either autonomic or neuropsychiatric issues, respectively, but this needs to be clarified in further longitudinal studies.

Finally, the logistic regression showed that total UPDRS III, Sexual disturbances and Acting out during dreams were the best explanatory variables, accounting for 50% of variance. However, caution is required in interpretation of these results, because two out of these three variables (i.e. UPDRS III and Sexual disturbances) have been used to generate the clusters. Nevertheless, these results might suggest that two main axes (motor and non-motor) should be used to clinically classify PD patients, playing such demographic features as age and gender a minor role.

We acknowledge that our study has some limitations. First, our cohort is unlikely to be representative of the whole parkinsonian population due to the recruitment performed in a tertiary care. Presumably, as patients referring to the tertiary care are usually younger [46], this type of recruitment is responsible for the lower age at onset as compared to naturalistic, community-based cohorts [47]. It may be also argued that with such follow-up (i.e. 2 years after the enrollment) some of our patients may have an atypical parkinsonism masquerading PD. We obviously can not definitively rule out the chance that someone can still convert into a diagnosis other than PD over the long-term period, but this would be unlikely. Indeed, mean disease duration of our cohort at the last examination was 36.8±7.6 months. This means that we are dealing with a three-year span, during which it would be very unlikely for an atypical syndrome to mimic a pure PD without any atypical sign. It would furthermore involve a very small percentage of patients (5% of patients had been already excluded for this reason as stated in the Results section) to interfere with the statistical power and overall interpretation.

Another exclusion criteria of ours, was treatment with antidepressant or anxiolytic drugs, and this might have lead to underestimation of depression and anxiety in our cohort. However, this issue should have played a minor role. Indeed, prevalence of depression and anxiety in our cohort was 45% and 54%, respectively, in line with previous reports [33].

Finally, our neuropsychological data were limited, thus hampering comparisons with previous studies focused on cognition in PD. It has been indeed unveiled that there is heterogeneity also when looking at cognitive performances in an incident PD cohort, being posterior cognitive deficits the strongest predictor for future development of dementia [48], [49].

In conclusion, the identification of these subgroups ought to serve more as a model for testing hypotheses, rather than as a definitive classification system, which should require final clinical-pathological data correlation. The existence of these subgroups needs of course further validation on independent cohorts of patients. Because our research project is ongoing and further publications are in preparation, data cannot yet be made widely accessible. However, we would be pleased to collaborate with other teams in the field. Researchers are encouraged to contact either the first author (erro.roberto@gmail.com) or the corresponding author (pbarone@unisa.it) with suggestions for collaboration or data sharing.
